# Evaluation of the Healing Effects of *Hypericum perforatum* and Curcumin on Burn Wounds in Rats

**DOI:** 10.1155/2020/6462956

**Published:** 2020-02-13

**Authors:** Nevra Seyhan

**Affiliations:** Department of Plastic Reconstructive, Aesthetic Surgery Gulhane Education and Research Hospital, Ankara, Turkey

## Abstract

**Background:**

For centuries, medicinal plants have been extensively used in wound healing of burn injuries. The aim of this study is to analyze comparatively the effects of curcumin and *Hypericum perforatum* (HP) on second-degree burn wounds in rats. *Materials and Methods*. This experimental study was conducted on 24 male Sprague-Dawley rats with second-degree burns. The animals were randomly divided into three groups. The burns were treated with curcumin (Group B) and *Hypericum perforatum* (HP) on second-degree burn wounds in rats.

**Results:**

All histological parameters of the control group showed statistically significant difference than the other groups (*p* < 0.05). There was no statistically significant difference between Groups B and C in terms of reepithelization and inflammation (*p* < 0.05). There was no statistically significant difference between Groups B and C in terms of reepithelization and inflammation (*p* < 0.05). There was no statistically significant difference between Groups B and C in terms of reepithelization and inflammation (*p* < 0.05). There was no statistically significant difference between Groups B and C in terms of reepithelization and inflammation (

**Conclusion:**

Both curcumin and *Hypericum perforatum* oil are effective in burn wound healing. Our findings showed a better quality of healing in curcumin-treated rats.*Hypericum perforatum* (HP) on second-degree burn wounds in rats.

## 1. Introduction

Burn injury is one of the most health-threatening problems in the world. Burn wound healing is a complex process including inflammation, granulation, and remodeling of the tissue. Plants have been used as therapeutics since ancient times [1]. Medicinal and traditional plants are considered as safe, natural, and inexpensive source of treatment for a wide variety of diseases. A wide variety of them have been reported to be useful in the treatment of burn wounds [2].

Turmeric, *Curcuma longa*, is an ancient spice used as a condiment. Curcumin is a component of the oriental spice turmeric that has been shown to have antioxidant and antiapoptotic properties. Curcumin, the natural yellow pigment in turmeric, is isolated from the plant *C. longa* [3]. *Hypericum perforatum* (St. John's wort) is a reputed plant with a long service to humankind. Extracts of St. John's wort contain many polyphenols including flavonoids, phenolic acids, naphthodianthrones and phloroglucinols.

It has been known that *Hypericum perforatum* [4] and curcumin [5] promote burn wound healing, but which one is more effective on wound healing has not been studied until now. The purpose of this study was to evaluate and compare the effects of curcumin and *Hypericum perforatum* on histological healing rates on burn wounds in a rat model.

## 2. Materials and Methods

We used 24 Sprague-Dawley rats (average weight 250–300 g, average age 3-4 months). The animals were obtained from kobaydeneyhayvanları san tic AŞ. They were divided into 3 three equal groups of 8 animals each; control (Group A), curcumin treatment (Group B), and *Hypericum perforatum* treatment (Group C) groups. They were all maintained in a sheltered environment (temperature: 20–25°C and humidity: 65–75%) under the supervision of a veterinarian. Animals were allowed free access to water and rat chow. The study protocol was approved by the local ethics committee with the approval number 371/2019.

**Figure 1:**
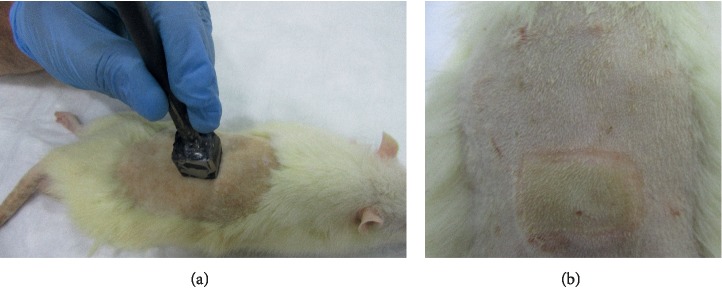
(a) The designed instrument was heated in boiling water for 5 minutes and was put on the shaved area to generate a burn wound. (b) Burn wound 2 × 2 cm diameter created on the back of the rat is seen.

The rats were sedated by intraperitoneal injection of ketamine (50 mg/kg) and xylazine (10 mg/kg), their back hairs were shaved using a razor, and the skin was cleansed with povidone iodine solution and then wiped with sterile water before induction of experimental burn injuries. A deep second-degree burn wound was created by using an iron hot plate (diameter 2 × 2 cm) warmed 5 minutes within boiling water and placed for 20 seconds on skin with pressure (Figures 1(a) and 1(b)).

Treatment began 24 hours after the burn injury. In Group B, curcumin oil (2 cc) and in Group C, *Hypericum perforatum* oil (2 cc) was applied once daily for 20 days. Group A was considered as the control group and received no medication.

At the end of the experiment (day 21), all rats were sacrificed with an overdose of anesthetics and burned surface areas were removed for histopathological examinations. Tissue samples were fixed in 10% neutral formalin. Tissues were embedded to paraffin wax, and sections were cut into 5 *μ*m thickness and stained with hematoxylin and eosin. Histologic parameters, epithelialization, granulation tissue formation, inflammation, and angiogenesis were assessed on biopsy specimens of the wound at the end of the study. At a magnification of ×40, histologic scores were made from 20 random fields per section from each specimen. The histologic scoring system ranged between 0 and 3 (Table 1).

### 2.1. Statistical Analysis

Collected data were analyzed using the Statistical Package for the Social Sciences (SPSS21). The values were evaluated as mean ± SD. Dual comparisons between groups exhibiting significant values were evaluated with the Mann–Whitney *U* test. *p* values less than 0.05 were considered as statistically significant.

## 3. Results

**Figure 2:**
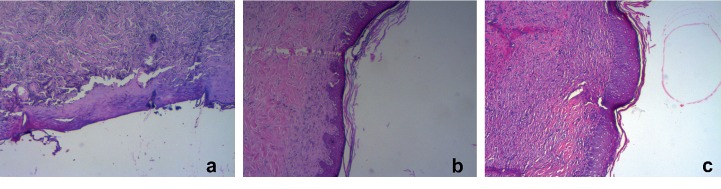
Histological epithelization (×40). Beginning of reepithelization in control group (a) and complete epidermal epithelialization in groups (b) and (c) is observed.

**Figure 3:**
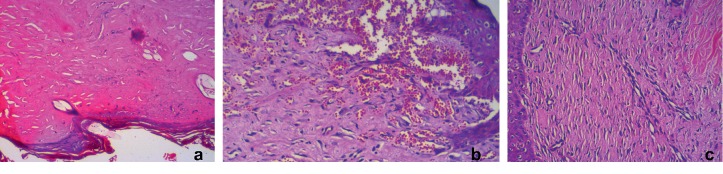
Histological neovascularization (×40). Marked angiogenesis is observed in curcumin-treated rats.

**Figure 4:**
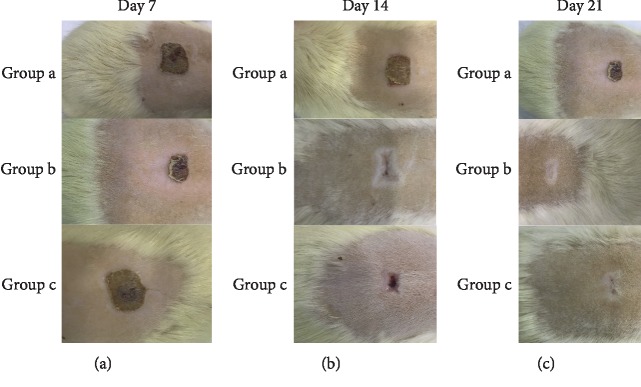
The macroscopic appeareance of the burn wounds on days (a) 7, (b) 14 and (c) 21.

**Figure 5:**
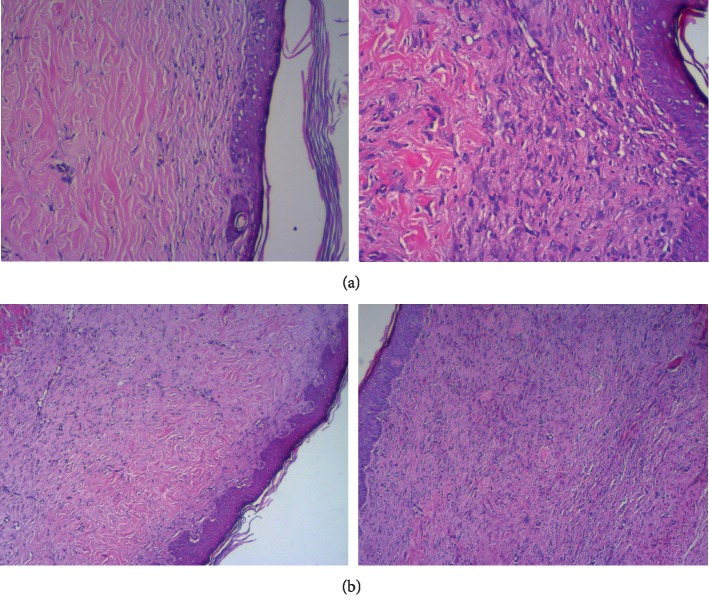
(a) Mild inflammation. (b) Dense fibrosis is seen in curcumin (group B) and *Hypericum perfotarum* (group C) groups.

On the 21st day, reepithelization was complete in curcumin- and *Hypericum perforatum*-treated groups, whereas in the control group, the epidermis layer was not formed (Figure 2). The curcumin group revealed remarkable healing with decreases in inflammatory cells with increases in fibroblast proliferation and angiogenesis. Neovascularization was significantly higher in curcumin-treated rats (Figure 3 and Table 2). On the basis of the taken photos at the 7, 14, and 21 days of the experiment, the quality of wound healing was better in the curcumin group (Figure 4). Thickness of granulation tissue was significantly different between each group (*p* < 0.0001). The mean values of thickness of granulation tissue in the center of the wounds for curcumin, *Hypericum perforatum* and control groups are shown in (Table 3). In the curcumin group, the granulation tissue was more organized. The number of inflammatory PMN cells was reduced, and the density of fibroblasts was increased (Figures 5(a) and 5(b)).

There was no statistically significant difference between Groups B and C in histopathological scores in terms of epithelial regeneration and inflammatory cells except angiogenesis (*p*=0.351, *p*=0.067 and *p*=0.045) respectively. A statistically significant difference was observed in Group A (control) when compared with other groups (B and C) regarding all scoring parameters (*p* < 0.05).

## 4. Discussion

Burn injuries constitute an important public health problem inducing numerous potentially fatal complications and impairing a person's psychological, social, physical functioning, aesthetic appearance, and quality of life [6]. The main causes of second-degree burns are scalds from hot water and liquids [7].

A growing number of scientific concerns are focusing on the significance of natural compounds that can act as wound healers. Phytomedicines to cure burns are brought into the medical forefront during the last decades. Numerous studies have examined effect of different medicinal herbs on the treatment of burn wounds. *Camellia sinensis* [8], *Arnebia euchromia* [9], nettle extract [10], licorice [11] were shown to be effective in burn wound healing.

Burn injury produces a burst of free radicals that increases oxidative stress in the cells [12]. Studies have shown that curcumin possesses many biological actions, including anti-inflammatory [13], anticancer [14], antioxidant [15], wound healing [16], and antimicrobial effects [17]. Curcumin is a polyphenolic compound, i.e., a mixture of three compounds, namely, diferuloyl methane, bisdemethoxycurcumin, and demethoxycurcumin,. Curcumin is a potent scavenger of free oxygen radicals [18]. The diferuloyl methane part of curcumin has antioxidant and anti-inflammatory properties without toxicity even at high doses [19].

St. John's wort, known botanically as *Hypericum perforatum*, is an important medicinal plant with diverse bioactive constituents such as naphtodianthrones, acyl-phloroglucinols, flavonoids, and xanthones, which have been reported to have anti-inflammatory, antimicrobial, antitumoral, antidepressant, and wound-healing activities [20, 21]. *Hypericum perforatum*'s main ingredient, hyperforin, was shown to activate the TRPC6 channel which had been recognised as an activator of keratinocyte differentiation [22].

*Hypericum perforatum* (St. John's wort, Kantoron) is a Turkish medicine used for treatment of many disorders and for pediatric nocturnal incontinence and as pain reliever, tranquilizer, and parasite-lowering ulcer healing agent [23].

The final step of the proliferative phase is epithelialization, which involves migration, proliferation, and differentiation of epithelial cells from the wound edges to resurface the defect. In this experimental study, we compared the effects of HP and curcumin on the burn wound healing process. Delayed burn wound healing was observed histopathologically in the control group while the epidermis layer was completely formed in curcumin- and *Hypericum perforatum*-treated groups. The formation of well-vascularized granulation tissue in the wound bed is a prerequisite for wound healing. Granulation tissue provides a stratum for epidermal cells to migrate and cover the wound. Curcumin contributed to healing by increasing vascularized granulation tissue. The median angiogenesis was higher in the curcumin-treated group when compared to control and *Hypericum perforatum*-treated groups.

Among antimicrobial agents, topical ointment of silver sulfadiazine is the most commonly deployed for partial- and full-thickness burns. However, SSD cream causes some systemic complications including neutropenia, erythema multiforme, crystalluria, and methemoglobinemia. For this reason, SSD is not recommended to be used for long periods of time [24]. Therefore, there is a need for more effective alternative burn dressings for burn patients with less adverse effects [25]. Natural products can be considered as an alternative source of treatment of burn wounds.

In the treatment of burns, the aim is to prevent infections and achieve the best functional aesthetic results in a shorter time with lower costs. Herbal oils as a suitable substitute for dressing and healing of burn wound injuries may be recommended. However, to clinically use these natural products, more supportive trials are needed. Although herbal products are extensively preferred and have become more widely available commercially, modern scientific methods and clinical trials are needed for confirming claims about their therapeutic effects.

## 5. Conclusion

With this study, we aimed at comparatively analyzing the effects of *Hypericum perforatum* and curcumin on wound healing in a burn wound model.

Both curcumin and *Hypericum perforatum* oil are effective in burn wound healing. Our findings showed a better quality of healing in curcumin-treated rats.

## Figures and Tables

**Table 1:** Scoring system of the histological changes in burn wound healing.

Groups	Epithelial regenaration	Inflammatory cells	Angiogenesis
Control	0.51 ± 1.12	2.72 ± 1.58	1.06 ± 0.12
Curcumin	2.69 ± 1.57	1.18 ± 1.02	3.32 ± 4.76
*Hypericum perforatum*	2.33 ± 1.64	1.26 ± 1.03	2.13 ± 3.84
*p*	*p*=0.351	*p*=0.067	*p*=0.045

**Table 2:** Histopathological evaluation of wound tissue samples.

Groups		*p*
Control	1.063 ± 0.02	0.001
Curcumin	2.493 ± 0.03	0.001
*Hypericum perforatum*	1.724 ± 0.02	0.001

**Table 3:** Thickness (mm) of granulation tissue in the center of the wound.

Score	Epithelialization	Inflammatory cells (PMN)	Angiogenesis
0	Absent	>40	None
1	Starting	20–40	Mild (<5 HPF)
2	Incomplete	10–20	Moderate (6–10 HPF)
3	Complete	0–10	Evident (>10 HPF)

## Data Availability

All data generated or analyzed during this study are included within this article.
